# *Tribulus terrestris* Efficacy and Safety Concerns in Diabetes and Erectile Dysfunction, Assessed in an Experimental Model

**DOI:** 10.3390/plants10040744

**Published:** 2021-04-10

**Authors:** Ruxandra Ștefănescu, Lenard Farczadi, Adina Huțanu, Bianca E. Ősz, Marius Mărușteri, Ancuța Negroiu, Camil E. Vari

**Affiliations:** 1 Department of Pharmacognosy and Phytotherapy, Faculty of Pharmacy, George Emil Palade University of Medicine, Pharmacy, Science and Technology of Targu Mures, 540142 Targu Mures, Romania; ruxandra.stefanescu@umfst.ro; 2 Center for Advanced Medical and Pharmaceutical Research, George Emil Palade University of Medicine, Pharmacy, Science and Technology of Targu Mures, 540142 Targu Mures, Romania; adina.hutanu@umfst.ro; 3 Department of Laboratory Medicine, George Emil Palade University of Medicine, Pharmacy, Science and Technology of Targu Mures, 540142 Targu Mures, Romania; 4 Department of Pharmacology and Clinical Pharmacy, George Emil Palade University of Medicine, Pharmacy, Science and Technology of Targu Mures, 540142 Targu Mures, Romania; bianca.osz@umfst.ro (B.E.Ő.); camil.vari@umfst.ro (C.E.V.); 5 Department of Medical Informatics and Biostatistics, George Emil Palade University of Medicine, Pharmacy, Science and Technology of Targu Mures, 540142 Targu Mures, Romania; marius.marusteri@umfst.ro; 6 Faculty of Pharmacy, George Emil Palade University of Medicine, Pharmacy, Science and Technology of Targu Mures, 540142 Targu Mures, Romania; negroiuanca@yahoo.com

**Keywords:** *Tribulus terrestris*, steroidal saponins, diabetes, erectile dysfunction

## Abstract

The present project aims to evaluate *Tribulus terrestris* (TT) extracts by addressing various possible mechanisms of action in order to see whether the use of TT supplements in diabetes and diabetes complications is justified. Diabetic rats were divided into three groups: diabetic control group, TT extract with low protodioscin content group (TT-LPC) and TT extract with high protodioscin content group (TT-HPC). After twelve weeks of treatment, fasting blood glucose, insulin, LH, FSH and testosterone levels were measured. Both TT preparations reduced elevated blood glucose level. Insulin and luteinizing hormone levels were not significantly different compared with the control group; however, the FSH and testosterone levels were significantly higher in the TT-HPC group compared with the diabetic control group. The testosterone level is correlated in part with the protodioscin concentration in extracts and is probably mediated through an FSH-linked pathway.

## 1. Introduction

*Tribulus terrestris* (TT) is one of the most controversial plants encountered in the literature. Several studies suggest that the administration of extracts obtained from the fruits could improve sexual performance, improve the symptoms of erectile dysfunction and increase libido in animals and/or humans [1,2]. On the other hand, there is the belief that the administration has no effects on the sexual parameters. Supposedly, these extracts could increase athletic performance, but the scientific results seem to be in contradiction [2,3]. The presumed actions are attributed to the high content in steroidal saponins, which are taught to have anabolic effects.

Data from the literature attributes supposed endogenous testosterone secretion to steroidal saponins based on favoring pulsatile LH release. However, effects like increasing testosterone levels in the body and the appearance of muscle hypertrophy, were observed mostly in preclinical studies and less in clinical ones: rodent experiments have shown rat performance increase in intense training and elevations in testosterone serum levels, but clinical trials conducted so far have had contradictory results [4,5].

Erectile dysfunction in diabetes has a multifactorial etiology, including metabolic, neurological, vascular and psychological components and, unfortunately, it is more resistant to treatment than erectile dysfunction of non-diabetic patients [6].

The main purpose of this experiment was to observe the effect of TT extracts on reproductive parameters in diabetes. A positive result could have a favorable impact on male diabetics, with a decrease in libido, testosterone production and inhibition of spermatogenesis. Secondary, other mechanisms of actions that can improve diabetic complications were noted.

The objectives of this study were to (i) quantify the protodioscin (PRD) content in the samples, (ii) to evaluate the antihyperglycemic effects of the extracts and (iii) to observe the effects of TT extracts on diabetic complications.

## 2. Results

### 2.1. LC-MS/MS Quantification of Protodioscin

In the first herbal drug (TT-1.2-17), the concentration of protodioscin was under the limit of quantification (<0.5 μg/g sample). This product was used in the experimental determination as the *Tribulus terrestris* low protodioscin content extract (TT-LPC).

The second herbal drug contained 162.42 µg/g protodioscin and was used in the experimental procedure as the *Tribulus terrestris* high protodioscin content extract (TT-HPC).

### 2.2. In Vivo Experiment

Forty-eight hours after STZ injection, rats exhibited diabetes-characteristic symptoms such as polydipsia and polyuria, notable for excessive water consumption and marked urination.

#### 2.2.1. Blood Glucose Variation during the Experiment

In the healthy group, the blood glucose level remained constant for 12 weeks. Blood glucose level significantly raised in all groups after STZ injection (week 1). After the beginning of the treatment (week 2), significant differences were noticed in TT-HPC group compared with the first week. As a response to the treatment, a statistically significant lower glucose level in the TT-LPC group was noticed in the fourth week compared with the first week. In this data analysis, the values from week 0 were used as control in the same group (Table 1).

#### 2.2.2. Weight Variation during the Experiment

Post-hoc testing revealed a significant increase in body weight in the TT-HPC group starting from the fourth week (Table 2). In the TT-LPC group, we noticed an increase in body weight in the first week compared with the initial weight, but in the second week, a decrease was recorded. At the end of the experiment, the mean body weight in the healthy group was 421 ± 34.79 g. In this data analysis, the values from week 0 were used as control in the same group.

#### 2.2.3. Oral Glucose Tolerance Test (OGTT)

OGTT was performed after 16 h of fasting in the 8^th^ week of the experiment. As seen in Table 3, after chronic administration, basal glycemia was influenced by the protodioscin content of the extracts and it was smaller in the TT-HPC group and higher in the TT-LPC group. However, no significant differences could be noted between groups after the administration of D-glucose solution (2.0 g/kg bw). In all groups, peak glucose levels were observed at 30 min and returned to the initial value at 90–120 min. These data suggest a potential protective effect of TT-HPC extract in streptozotocin-induced diabetes in chronic treatment without having a direct influence on normalizing blood glucose levels after glucose loading.

#### 2.2.4. Sperm Morphology

Modifications in semen morphology (coiled tail, missing tail, micro-head and abnormal shape) were noticed in every group, with high frequency in the DC group (Table 4). In the TT-HPC and TT-LPC groups, a reduction of abnormal shape germ cells was observed compared with the DC group. In the healthy group, the percent of normal cells was 83.13 ± 6.11. Even though there were no significant statistical differences between groups, the potential benefits of *Tribulus terrestris* extracts in alleviating germ cells destruction in diabetes should be considered. 

#### 2.2.5. Insulin, LH, FSH

**Figure 1:**
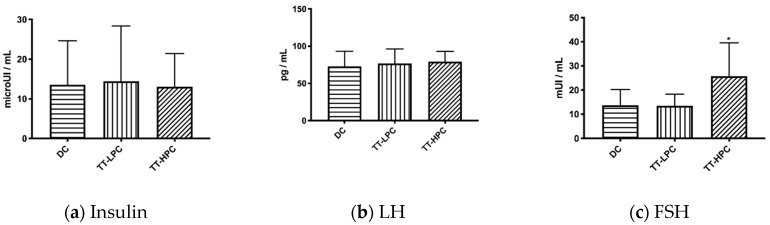
Biochemical parameters; bars represent means ± SD for ten rats in each group for LH and FSH; for insulin in DC group n = 8, in TT-LPC group n = 9 and in TT-HPC group n = 8; * represents significant differences compared with DC and TT-LPC groups (*p* < 0.05).

As shown in Figure 1, the insulin level was not significantly different between the diabetic groups. As expected, the insulinemia was higher in the healthy group (29.73 ± 7.27 micro UI/mL). Luteinizing hormone levels were not different between the groups, all groups having a LH level similar to the mean value determined in the healthy group (75.52 ± 23.41 pg/mL). On the other hand, significant differences were observed in foliculo-stimulating hormone levels in the DC and TT-LPC group compared with TT-HPC group. FSH level in the healthy group was 30.79 ± 7.29 mUI/mL, similar with the results obtained for TT-HPC group.

#### 2.2.6. Testosterone Levels 

**Figure 2:**
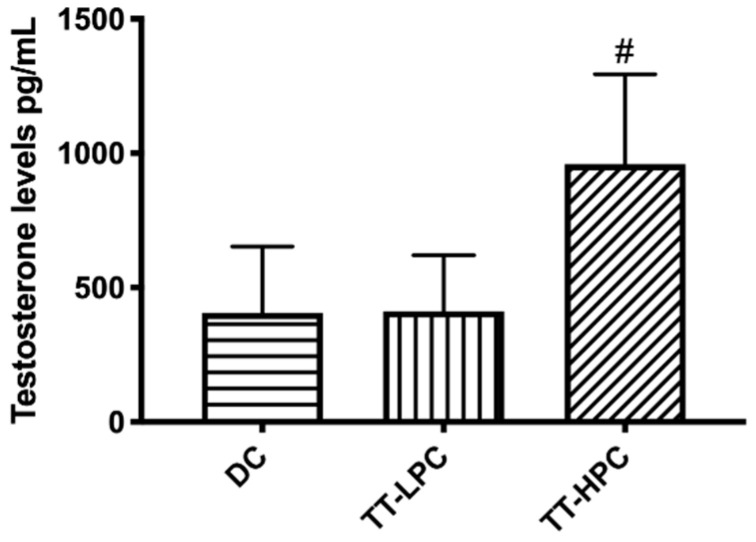
Testosterone levels in the experimental groups. Bars represent mean ± SD for eight rats in each group; bars marked with; # represents significant differences compared with DC and TT-LPC groups at (*p* < 0.01).

Testosterone levels were significantly lower from a statistical point of view in the diabetic control group and in the group treated with an extract with low protodioscin content compared with TT-HPC group (Figure 2). The normal level of testosterone in the healthy group was 1119 ± 468.2 pg/mL.

#### 2.2.7. Opacity Grading of Lenses 

**Figure 3:**
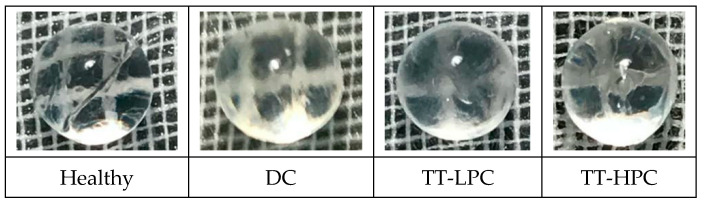
Photos of lenses from each group.

A stage 3 or 4 of opacity was not observed in any of the groups (Figure 3). No significant differences were noted between the four groups regarding the formation of cataract.

## 3. Discussion

Different studies on the efficiency of TT administration have highlighted the existence of major discrepancies in terms of treatment efficiency. It is largely documented how pedo-climatic conditions influence the chemical composition of herbal drugs [7] to a great extent. 

Prior to our experiment, an evaluation of TT supplements found in pharmacies was conducted [8]. For this experiment we randomly selected an herbal drug with very low protodioscin content and one with high protodioscin content, in order to evaluate the influence of PRD on the treatment outcome, starting from the assumption that this compound could be partially responsible for the documented effects of TT. 

The control of postprandial blood glucose concentrations is critical for the management of diabetes. Most of the diabetic complications are associated with uncontrolled glycemia through the activation of different pathways: polyol, advanced glycation end products (AGEs), protein kinase C, hexosamine pathway and enediol [9].

The diabetogenic activity of STZ is caused by its preferential accumulation in β-cells through the GLUT2 transporter system resulting in cytotoxicity; therefore, STZ-induced diabetes is an animal model that reproduces type I diabetes and its complications, including the effects on the male reproductive system [10]. 

The mechanism by which the antihyperglycemic effect of *Tribulus terrestris* appears is unknown, but recent studies suggest that this action could be related to the ability of TT to inhibit α-glucosidase and α-amylase [11]. Although this mechanism cannot be ignored, fasting blood glucose level is rarely influenced by alpha-glucosidase and alpha-amylase inhibitors. These compounds act specifically in reducing or even preventing post-prandial hyperglycemia. As a result of TT administration, rat blood glucose decreased not only in the case of the TT-LPC, but, especially in the case of the TT-HPC, with a significant difference in the twelfth week compared with the first week.

A weight loss was observed after the first week in TT-LPC and TT-HPC groups, but we consider it to be normal, given that this experimental model is of type I diabetes.

In the case of TT-HPC, there was a marked decrease in fasting blood glucose from the first week of treatment, as opposed to TT-LPC where the decrease was slow, the lowest value being recorded only after 8 weeks of treatment. The efficacy of *Tribulus terrestris* extract in normalizing blood glucose, both basal and postprandial, has already been reported in diabetic rabbits. An improvement in insulinemia was also observed in these studies [12]. The effect of *Tribulus terrestris* on glucose level may be the result of increased alpha-glucosidase and aldose-reductase activity, suggesting that the action is antihyperglycemic and not hypoglycemic. The antihyperglycemic effect has been attributed to saponins, especially to protodioscin [1].

The marked decrease of fasting blood glucose in the case of TT-HPC compared with TT-LPC observed in our study, could be determined by the differences in the concentration of the protodioscin, but not by its antihyperglycemic action. Although protodioscin was below the limit of detection in the analytical method used, a decrease in fasting blood glucose was also observed in this case, the differences being significantly different between the values determined in the first week and those at the end of the experiment. However, the decrease was slower, only in the eighth week were the values lower than those found in the DC group. Because, in this case, the effect cannot be correlated with the activity of protodioscin, we intended to see if the effect is hypoglycemic rather than antihyperglycemic. This type of action has been reported in the scientific literature, being proposed as a mechanism for the proliferation of pancreatic beta cells, with the consecutive increase of insulin secretion and stimulation of peripheral glucose utilization [13,14]. This effect can be attributed to flavonoids (kaempferol and quercetin), since long-term administration of quercetin has been proved to normalize glycemia in streptozotocin-induced diabetic animals [15]. Unexpectedly, following the determination of insulinemia, no statistically significant differences were observed between DC, TT-LPC and TT-HPC groups (Figure 1) which excludes such a mechanism. 

Administration of TT extracts is believed to be responsible for the increase of testosterone or testosterone precursors in the blood, which explains the increasing number of nutritional supplements available on the market. The anabolic actions of *Tribulus terrestris* herbal drugs are attributed to their content in steroidal saponins, mainly furostanol saponins. Experimental diabetes has been described in the literature to produce changes in the male reproductive system [6]. It has been noticed that male diabetic rats have significantly lower levels of testosterone compared with healthy rats. It was clear from the present study that this type of diabetes significantly impaired testosterone production since testosterone levels in the diabetic control group were reduced with almost 64%, compared with the values determined in healthy rats. A bidirectional relationship seems to exist between serum insulin and testosterone. Although testosterone levels were significantly lower in the DC and TT-LPC groups, LH levels (luteinizing hormone that controls Leydig cell function and testosterone production) were not different between the experimental groups [16]. Testosterone has a negative feedback on the production of LH. When testosterone levels are low, there is a compensatory increase in LH which if not accompanied by a consecutive increase in testosterone suggest a primary failure of Leydig cells.

The positive effects of TT on testosterone and sexual dynamics have been attributed to steroidal saponins, but polyphenolic content must not be ignored because these compounds play an important role in chronic conditions due to their antioxidant capacity [17].

Studies with animal models of STZ-induced diabetes have shown that both type 1 and type 2 DZ alter male fertility by modifying sperm specific parameters (sperm motility, sperm DNA integrity and sperm morphology) [18,19]. These changes may occur as a result of impaired spermatogenesis, but also due to inhibition of intracellular glucose transport as a result of insulin deficiency. Glucose is used for energy purposes and is essential for providing the energy needed to maintain motility [20]. One of the objectives of our study was to evaluate a possible beneficial effect of *T. terrestris* extracts in improving the negative impact of diabetes on sperm morphology. Interestingly, an improvement in sperm morphology was observed in both groups treated with TT extracts. However, in the TT-HPC group, testosterone and FSH levels were significantly higher than those in the TT-LPC group. In addition, these results can be explained by the differences in the concentration of protodioscin, structurally similar to DHEA (dehydroepiandrosterone), a precursor of testosterone. Therefore, administration of a protodioscin-rich extract explains an increase in plasma testosterone levels observed in the TT-HPC group. Male infertility is associated with sperm DNA fragmentation as a result of an apoptotic process or the destruction of cells by ROS (reactive oxygen species). Impairment of the integrity of the DNA may occur in the testis, epididymis, deferential vessels or after ejaculation. In diabetes, this degradation of DNA was associated with increased ROS production in seminal plasma [21].

Oxidative stress occurs when the balance between reactive oxygen species and antioxidants is inclined towards the formation of free radicals. Secondary metabolites of TT are known to have antioxidant properties. Although the content in polyphenols is lower than in other herbal preparations, the antioxidant action of *Tribulus terrestris* is not to be ignored [17]. The advantage of this plant and its phytocomplex is that the presence of saponins in the extract improves the solubility of the polyphenolic compounds and the in vivo absorption [22]. Polyphenolic compounds found in TT are mainly glucosides of quercetin and kaempferol and quinic acid derivatives. Numerous studies have shown that these polyphenolic compounds have a wide range of activities and the antioxidant capacity is among them [23,24].

Although the antioxidant action of TT has already been studied and demonstrated, a more plausible explanation of the superior effect of TT-HPC in reducing the negative effects of diabetes on morphological parameters could be attributed to FSH growth. The direct anti-apoptotic action of FSH on germ cells and also the inhibition of DNA fragmentation has been demonstrated in both preclinical and clinical studies [25].

The high differences noticed between the two herbal drugs are presumably related to the protodioscin content. 

Overall, the results obtained are in correlation with other recently published studies that have shown that TT administration in male rats has positive effects on sexual parameters but also on plasma levels of gonadotropins and testosterone [26]. Our results are in accordance with Ballester et al. and suggest that there is a connection between diabetes and spermatogenesis and the mechanism is mainly connected with the FSH levels [16]. This suggests that the FSH-mediated pathway plays an important role in testosterone secretion.

As shown in our previous study, STZ-induced diabetes can, in time, lead to the development and progression of cataract in experimental animals. However, in this study, no signs of early cataract development were observed in diabetic animals. The difference between our studies could be related to the gender dimorphism [27]. In our previous work, the study design was based on female Wistar rats. Some reported studies have highlighted the important role of gender in the evolution of diabetes and diabetic complications [28,29]. Since some studies have reported that also diabetic male rats developed cataract, we can only assume that the age of the rats could be an important factor in the progression of diabetes-related complications. For example, Bahmani et al. [30] and Suryanarayana et al. [31] used younger rats in their studies. In addition, according to Wang-Fisher et al. [32], cataract development is time-dependent and they reported a low percent of rats with cataract in the first 15 weeks after STZ injection.

## 4. Materials and Methods

### 4.1. Materials

Two different *Tribulus terrestris* herbal drugs (fruits) were purchased from a local pharmacy and were identified at the Department of Pharmacognosy and Phytotherapy, Faculty of Pharmacy, George Emil Palade University of Medicine, Pharmacy, Science and Technology of Targu Mures, Romania. A voucher specimen of each sample (TT-1.2-17 and TT-2.2-17) was archived at the same department. 

Protodioscin was purchased from Cayman chemical, Testosterone-2,3,4-^13^C_3_ from Sigma-Aldrich Corp. (St. Louis, MO, USA), testosterone from AppliChem. All the other used reagents were of analytical grade.

### 4.2. Sample Preparation

Both samples were extracted twice for 60 min, with 70% ethanol, in an ultrasonic water bath. The ethanol was evaporated in a Rotary evaporator and the extracts were concentrated until each had a concentration in total saponins of 20% (gravimetric determination, as previously described [8]). The extracts were stored at −20 °C until analysis and administration.

### 4.3. LC-MS/MS Quantification of Protodioscin

LC MS/MS analysis was performed using a QTOF 4600 (AB Sciex), UHPLC Flexar FX-10 (Perkin Elmer) following a previously described method [33]. Chromatographic separation of protodioscin was performed on a C18 column, with a mixture of 1 mM ammonium acetate buffer (phase A) and acetonitrile (phase B) as a mobile phase. The analysis time was 10 min for the samples and 3 min for the standard solutions.

Ionization of analytes was performed in electrospray negative mode, whereas protodioscin detection was performed by monitoring the sum of ions *m/z 737.41, m/z 739.42* and *m/z 755.42* resulted from *m/z 1047.7* at a collision energy of 65V.

### 4.4. In Vivo Study Design

Prior approval was received from the ethical committee of the university and from the Sanitary Veterinary National Authority. All experiments were conducted in compliance with the Arrive guidelines and in accordance with the EU Directive 2010/63/EU for animal experiments. According to the Guidance Document on Acute Oral Toxicity Testing based on oral LD50 value which were recommended by the Organization for Economic Cooperation and Development, the extract of TT may be assigned to be class 4 (LD50 > 300–2000 mg/kg body weight).

For this experiment, fifty male Wistar rats were used (between 5 and 7 months old). The animals were kept in standard conditions, in individual cages, with 12 h light/dark cycles. All animals were allowed one week of accommodation prior to the beginning of the study. During the experiment all animals received standard pellets and water ad libitum. Diabetes was induced by a single i.p. injection with 60 mg/kg streptozotocin (STZ). After 48 h, blood glucose was measured using a commercial glucometer (Accu-Check Active) and rats with blood glucose level higher that 250 mg/dL were considered diabetic and were included in the present study (ten rats were excluded from the present study). A homogenous suspension of the extracts (previously defrosted), in water, was prepared freshly every time prior to administration. Thirty animals were assigned following simple randomization procedures to three experimental groups of 10 animals each: diabetic control (DC), *Tribulus terrestris* extract with low protodioscin content group (TT–LPC)–25 mg extract/bw and *Tribulus terrestris* extract with high protodioscin content group (TT - HPC) – 25 mg extract/bw. An additional healthy group of ten rats was used in order to compare the evolution of healthy animals versus diabetic animals. This group was also used to obtain orientation values for different parameters measured under the study’s conditions (weight, insulin, testosterone, LH, FSH, sperm morphology, opacity grading of lenses), but these values were not included in the statistical analysis. The extracts (TT groups) or vehicle only (healthy and control groups) were administered through gavage. Glucose level, body weight and cataract development were monitored for twelve weeks. 

Oral glucose tolerance test (OGTT) was performed in the 8th week of the experiment after 16 h of fasting. Rats were orally dosed with a D-glucose solution (2.0 g/kg bw) and blood glucose concentrations were subsequently measured at 0 (just prior to oral glucose dosing), 30, 60, 90 and 120 min after the oral dosing of glucose.

Specific complications of diabetes were monitored during the experiment. At the end of the study, the rats were sacrificed by isoflurane anesthesia with no chances of recovery. The blood was collected in separate vials for each determination and centrifuged at 280× *g* for 10 min at 4 °C. The serum was separated and stored at −70 °C until analysis.

### 4.5. Sperm Morphology

After the sacrifice of the animals, the proximal caudal portion of epididymis was dissected and a sample of sperm was collected in order to evaluate the morphological characteristics [34].

We analyzed 200 spermatozoa for each slide and changes in the shape of the head and tail were noted.

The analysis was performed according to the WHO guidelines for human semen, adapted for the rat semen [35]. The following defects were monitored: macro- or micro-heads, double heads, abnormal shape of the head, abnormal insertion of the head, short tail, long tail, missing tail, broken, coiled or other abnormal shapes of the tail.

### 4.6. LH and FSH Determination

For both luteinizing hormone and follicle-stimulating hormone from serum, enzyme-linked Immunosorbent (ELISA) assay kits for *Rattus norvegicus* were used (Cloud-Clone Corp. for LH and Cusabio, CSB-E06869 for FSH, respectively. The ELISA protocols were run on the automated ELISA instrument (DSX Dynex Technologies, USA) according to the manufacturer’s instructions.

### 4.7. Testosterone Determination

An LC-MS/MS method was used to test testosterone from rat serum samples based on a modified method previously validated (manuscript under review). After extraction of the testosterone and testosterone (2,3,4-^13^C3) internal standard from the serum samples obtained from rats by a “high throughput” extraction method (protein precipitation), the resulting samples were analyzed by reverse phase liquid chromatography and detected following specific transitions in MRM MS/MS mode of the mass spectrometer after pre-ionization by a positive electrospray ionization source (ESI +). The calibration curve with testosterone was composed of 8 levels with concentrations of 50–10000 pg/mL. The calibration curve was plotted using a weighting factor of 1/y2 and the accuracy of each calibration standard was calculated. There were no standards excluded from the final curve due to the deviation from the theoretical value > 15%. Testosterone-2,3,4-^13^C_3_ was used as an internal standard.

Unknown serum samples were freshly prepared on the day of analysis and were placed in the auto sampler immediately after preparation. We added 250 μL of serum sample and 100 μL internal standard solution (10 ng/mL) to an Eppendorf tube after which 150 μL of 6% perchloric acid solution in acetonitrile was added. The mixture was vortexed for 2 min at 2000 rpm and then centrifuged at 10,000 rpm for 10 min. The supernatant was transferred to a HPLC vial to be injected into the LC-MS/MS system. 

### 4.8. Opacity Grading of Lenses

The lenses collected from each rat at the end of the experiment were analyzed immediately under the microscope. The lenses were analyzed according to the method described by Geraldine et al. and were classified into four grades: 1—no opacity, 2—minor opacity, 3—mild opacity and 4—severe opacity [36].

### 4.9. Statistical Analysis

Results were expressed as mean ± SD. Statistical analysis was performed using GraphPad Prism^TM^ for macOS (version 9.0.2, Graphpad software^®^, CA, USA). Normal distribution was confirmed by Kolmogorov–Smirnov test. One-way analysis of variance (ANOVA), followed by post-hoc Tukey test was used for the analysis of the results obtained at the insulin, testosterone, FSH and LH determinations. Two-way ANOVA followed by post-hoc Tukey test was used for the analysis of weight and glucose level variations during the study. Chi square test was used for the determination of association between groups and morphological changes of spermatozoa. Statistical analysis was undertaken only for studies where each group size was at least n = 8 (where hemolysis was present, those samples were excluded from the statistical analysis in order to avoid the interference with the measurements), the group size being the number of independent values not technical replicates. The results were considered significant if *p* < 0.05. Values obtain from the healthy group were not included in the statistical analysis.

## 5. Conclusions

Several mechanisms of action have been put forward in order to explain the effects of TT extract on diabetic rats and our results indicate that TT extracts have the potential to reduce hyperglycemia in diabetic rats. The extract with a high protodioscin content can also alleviate testosterone levels.

Pharmacokinetic data are still insufficient in order to properly evaluate the absorption, distribution, metabolism and elimination of the active compounds from TT extracts.

As such, further studies are required to evaluate the bioavailability of steroidal saponins in the human body in order to outline the clinical relevance.

## Figures and Tables

**Table 1:** Fasting blood glucose variations during the experiment.

Week	DC	TT-LPC	TT-HPC
Week 0	106 ± 5.7	114.25 ± 7.98	110.63 ± 7.78
Week 1	360.75 ± 89.8 ^†^	505.12 ± 95.85 ^‡^	432.75 ± 96.5 ^‡^
Week 2	378.3 ± 162.4 ^‡^	447 ± 83.38 ^‡^	167.88 ± 101.91
Week 4	302. 25 ± 144.4 *	380.62 ± 99.38 ^‡^	200.63 ± 168.53
Week 6	318.88 ± 131.5 ^#^	341.12 ± 107.08 ^#^	189.63 ± 142.69
Week 8	337.75 ± 76.6 ^#^	264.62 ± 97.72	195.63 ± 103.66
Week 10	289.75 ± 102.3 *	301.37 ± 118.65 *	253 ± 174.26
Week 12	354.5 ± 53.8 ^†^	279.12 ± 93.33	216.38 ± 132.47

Data are expressed as mean ± standard deviation. The superscript indicates a significant difference between the values from week 0 compared with the values from all other weeks, in the same group. * *p* < 0.05, ^#^ *p* < 0.01, ^†^ *p* < 0.001, ^‡^ *p* < 0.0001.

**Table 2:** Weight variations during the experiment.

Week	DC	TT-LPC	TT-HPC
Week 0	244 ± 43.3	253.75 ± 28.75	247.5 ± 28.6
Week 1	285 ± 45.5	329 ± 36.95 ^#^	310 ± 28.78 *
Week 2	332 ± 39.4 ^†^	282.5 ± 55.48	306.25 ± 37
Week 4	332 ± 37.7 ^†^	305 ± 24.49	320 ± 23.3 ^#^
Week 6	320 ± 44 ^#^	283.75 ± 32.04	338.75 ± 42.57 ^†^
Week 8	312 ± 47.3 *	278.75 ± 34.82	342.5 ± 43.01 ^‡^
Week 10	311.11 ± 45.1 *	297.5 ± 28.66	353.5 ± 52.01 ^‡^
Week 12	307.78 ± 43.2 *	286.25 ± 36.23	358.75 ± 32.7 ^‡^

Data are expressed as mean ± standard deviation. The superscript indicates a significant difference between the values from week 0 compared with the values from all other weeks, in the same groups. * *p* < 0.05, ^#^ *p* < 0.01, ^†^ *p* < 0.001, ^‡^ *p* < 0.0001.

**Table 3:** Oral glucose tolerance test.

Time (min)	DC	TT-LPC	TT-HPC
0	318.88 ± 131.5	341.12 ± 107.08	189.63 ± 142.69
30	364.5 ± 196.8	361.22 ± 118.08	213.7 ± 109.31
60	315.13 ± 155.32	350.22 ± 101.32	210.2 ± 117.49
90	326.75 ± 129.85	332.22 ± 104.47	183.2 ± 88.95
120	308.75 ± 81.49	280.37 ± 95.07	180.62 ± 86.4

Data are expressed as mean ± standard deviation.

**Table 4:** Normal cells in sperm morphology evaluation.

	DC	TT-LPC	TT-HPC
% normal cells	71 ± 6.41	81 ± 7.76	78.88 ± 6.98

Data are expressed as mean % ± standard deviation.

## Data Availability

Not applicable.
